# Distinguished Honor

**Published:** 1886-07

**Authors:** 


### EDITORIAL.

### Distinguished Honor.

It is, doubtless, a matter of gatification and pleasure to all true members of the dental profession, to know that a high honor has been conferred upon one of its members, one, too, for whom the universal suffrage of the profession of this and every other country would have been rendered, had the question been one open for such an expression.

John Tomes has been knighted by Her Majesty, England's sovereign, the second dentist of the world to receive such a distinction. The first was Sir Edwin Sanders, whom all were delighted to have thus honored. In certain lines of scientific and practical work, Sir John Tomes has done more than any man now living. It may be said that he has had great opportunities; but he has improved these as scarcely any one else would have done. It may, with equal truth, be said, that the period of his greatest labor was one in which there were great adverse environments. Embarrassments were met and overcome by him that the dentists of this day know little or nothing about.

The facilities for the prosecution of his work were meager indeed as compared with those of the present. He had not the stimulus and moral support in the recognition and appreciation of dentistry that now obtains.

In spite of difficulties, he achieved a reputation and position in his chosen profession second to none in his own or any other country.

He has done more than any one else to secure for the dental profession a legal recognition and status. He formulated, and had enacted, the best law for the regulation of dental practice that has as yet appeared.

Sir John Tomes has conducted the great work of his life without exciting any special antagonisms, prejudices, or jealousies, which is one of the best evidences of a great and good man.

The following, from the *London Lancet*, a medical journal of high standing, and always guarded in its statements, will be interesting to the dental profession as giving some matters of an historical nature, and also as an expression from one not connected with dentistry:

HONORS TO THE DENTAL PROFESSION.

The announcement that the Queen has been graciously pleased to confer the honor of knighthood on Mr. John Tomes will be received with great satisfaction, not only by the dental section, but by all branches of the profession. Sir John Tomes, who comes of an old Gloucestershire family, was apprenticed to a general practitioner at Evesham, and afterwards entered as a student at King's College and Middlesex Hospitals, at the latter of which he was house-surgeon for two years, and, by his energy and scientific attainments, won the esteem and sincere friendship of Sir Thomas Watson, Messrs. Henry Arnott and Campbell De Morgan, a friendship which only terminated with their lives, and it was chiefly by the advice of Sir Thomas Watson that he turned his attention to dental surgery. For some years he devoted a considerable portion of his time to the compilation of numerous papers on dental surgery and dental comparative anatomy, and in recognition of his original researches, was elected a Fellow of the Royal Society in 1852. To Sir John Tomes is due the gratitude of the dental profession for having prevailed upon the council of the Royal College of Surgeons to apply for a charter to enable them to grant a license in Dental Surgery, and in this he was greatly assisted by his friends Arnott, Lawrence, and Green, who were then members of the council. Sir John Tomes was one of the first examiners in connection with the new license, and also on the original staff of the Dental Hospital, London, which he largely helped to found. In 1862 he was presented with a testimonial, consisting of a silver tea and coffee service by his brother practitioners, "In acknowledgment of the many valuable services he has rendered to his profession." Some ten years ago, after a severe illness, he began to relinquish practice, and ultimately gave up altogether, but only to work all the harder for the interests of his profession. He was one of the most powerful promoters of the Dentist's Act, which he always looked upon as a natural sequence of the granting of a diploma by the college. In 1883, the honor of the Fellowship of the Royal College of Surgeons was conferred upon him at the same time as it was upon Prof. Huxley.

